# IgE to cyclophilin Bet v 7 triggers mast cell activation and mediates cross‐reactivity with Ara h 18 in children with seasonal allergic rhinitis

**DOI:** 10.1111/pai.70308

**Published:** 2026-03-04

**Authors:** Wilhelm Sponholz, Carolin Steinert, Francesco Monnati, Ekaterina Potapova, Jonas Lidholm, Lars Mattsson, Enrico Scala, Roberto Bernardini, Carlo Caffarelli, Antonella Casani, Rosa Cervone, Elisabetta Del Duca, Pasquale Comberiati, Giovanna De Castro, Michele Miraglia Del Giudice, Iride Dello Iacono, Andrea Di Rienzo Businco, Marcella Gallucci, Arianna Giannetti, Viviana Moschese, Elena Varin, Annamaria Bianchi, Mauro Calvani, Tullio Frediani, Francesco Macrì, Nunzia Maiello, Francesco Paravati, Umberto Pelosi, Diego Peroni, Giuseppe Pingitore, Mariangela Tosca, Anna Maria Zicari, Giampaolo Ricci, Riccardo Asero, Salvatore Tripodi, Paolo Maria Matricardi, Joerg Scheffel

**Affiliations:** ^1^ Institute of Allergology, Charité – Universitätsmedizin Berlin, Corporate Member of Freie Universität Berlin and Humboldt‐Universität zu Berlin Berlin Germany; ^2^ Fraunhofer Institute for Translational Medicine and Pharmacology ITMP, Immunology and Allergology Berlin Germany; ^3^ Department of Biology, Chemistry and Pharmacy Freie Universität Berlin Berlin Germany; ^4^ INFN & Department of Physics University of Rome Tor Vergata Rome Italy; ^5^ Department of Pediatric Respiratory Medicine, Immunology and Critical Care Medicine Charité – Universitätsmedizin Berlin, Corporate Member of Freie Universität Berlin and Humboldt‐Universität zu Berlin Berlin Germany; ^6^ Thermo Fisher Scientific Uppsala Sweden; ^7^ Clinical and Laboratory Molecular Allergy Unit, IDI‐IRCCS Rome Italy; ^8^ Pediatric Unit San Giuseppe Hospital Empoli Italy; ^9^ Clinica Pediatrica, Department of Medicine and Surgery University of Parma Parma Italy; ^10^ Pediatra di Libera Scelta Benevento Italy; ^11^ UOSD di Immunopatologia ed Allergologia Pediatrica, Policlinico Tor Vergata Università di Roma Tor Vergata Rome Italy; ^12^ Department of Clinical and Experimental Medicine, Section of Paediatrics University of Pisa Pisa Italy; ^13^ Pediatric Department La Sapienza University Rome Italy; ^14^ Dipartimento della Donna, del Bambino e di Chirurgia Generale e Specialistica Università della Campania Luigi Vanvitelli Naples Italy; ^15^ Pediatric Unit Fatebenefratelli Hospital Benevento Italy; ^16^ Pediatric Department and Pediatric Allergology Unit Sandro Pertini Hospital Rome Italy; ^17^ Pediatric Unit IRCCS Azienda Ospedaliero‐Universitaria di Bologna Bologna Italy; ^18^ Allergology Service San Carlo Clinic Paderno Dugnano, Milan Italy; ^19^ Pediatric Unit San Camillo Forlanini Hospital Rome Italy; ^20^ Pediatric Unit Crotone Italy; ^21^ Pediatric Unit Santa Barbara Hospital Iglesias Italy; ^22^ Pediatric Unit Grassi Hospital Rome Italy; ^23^ Pulmonary Disease and Allergy Unit Istituto Giannina Gaslini IRCCS Genoa Italy; ^24^ Ambulatorio di Allergologia Clinica San Carlo Paderno Dugnano, Milan Italy

**Keywords:** Ara h 18, Bet v 7, Component‐resolved diagnostics, Cyclophilins, IgE cross‐reactivity, Immunoglobulin E, Mast cell activation, Panallergens, Pollen–food allergy syndrome, Seasonal allergic rhinitis

## Abstract

**Background:**

Panallergens are ubiquitously expressed molecules that may drive IgE sensitization across diverse allergen sources, thereby shaping complex clinical phenotypes such as asthma and pollen–food allergy syndrome (PFAS). Although several panallergen families have been extensively characterized, unexplained sensitization patterns are still observed in clinical practice. Cyclophilins, a conserved protein family, have recently been proposed as candidate panallergens; however, their molecular, clinical, and functional roles remain largely undefined.

**Methods:**

Within the Panallergens in Pediatrics (PAN‐PED) cohort, we investigated 100 Italian children with seasonal allergic rhinitis (SAR) for IgE sensitization to the cyclophilins Bet v 7 and Ara h 18. Functional assays were performed by sensitizing mast cells with patient sera (*n* = 11) and assessing activation upon stimulation with increasing concentrations of recombinant Bet v 7 (rBet v 7), using CD63 expression as readout. Additional experiments evaluated the impact of heat treatment (60°C, 80°C, or 100°C) and saliva digestion on rBet v 7 activity.

**Results:**

rBet v 7 induced mast cell activation in a dose‐dependent manner, with peak responses at intermediate allergen concentrations, followed by attenuation at higher doses. rBet v 7 maintained functional activity after exposure to mild heat and saliva. A strong correlation was found between specific IgE levels to Bet v 7 and Ara h 18 (*r* = 0.996, *p* < 0.0001).

**Conclusion:**

Bet v 7 has the functional capacity to activate mast cells and is resistant to mild heat and saliva digestion, supporting its role as a clinically relevant panallergen. In addition, Bet v 7 shows high similarity to cyclophilins from other plant sources and exhibits virtually identical IgE‐binding properties to Ara h 18. These findings suggest a potential rationale for considering the inclusion of cyclophilins into molecular diagnostic panels, particularly in children with SAR.

**Abbreviations**
AIT: allergen immunotherapy
BAT: basophil activation test
CsA: cyclosporin A
DPBS: Dulbecco's Phosphate‐Buffered Saline
FSC: forward scatter
IMAC: immobilized metal ion affinity chromatography
IQR: interquartile range
MAT: mast cell activation test
nsLTP: non‐specific lipid transfer protein
PAN‐PED: Panallergens in Pediatrics cohort
PFAS: pollen–food allergy syndrome
PPIase: peptidyl‐prolyl cis‐trans isomerase
PR‐10: pathogenesis‐related protein family 10 members
PSCMCs: primary human mast cells from peripheral blood precursors
r: recombinant
RT: room temperature
SAR: seasonal allergic rhinitis
SD: standard deviation
SEC: size‐exclusion chromatography
sIgE: specific IgE
SPT: skin prick test
SSC: side scatter
tIgE: total IgE

**Key message**

Cyclophilins represent a previously underrecognized family of plant panallergens. This study demonstrates that IgE to birch pollen cyclophilin Bet v 7 can trigger mast cell activation and exhibits strong cross‐reactivity with peanut cyclophilin Ara h 18 in children with seasonal allergic rhinitis. Bet v 7 retains allergenic activity after mild heat and saliva exposure, underscoring its potential clinical relevance. These findings provide additional support for cyclophilins as functional panallergens and suggest their potential utility in molecular allergy diagnostic panels to improve characterization of complex sensitization patterns in pediatric allergic disease.

## 1 INTRODUCTION

Cyclophilins constitute a family of ubiquitous proteins that are remarkably conserved across evolution and present in organisms ranging from bacteria to humans, underscoring their functional importance.^1^, ^2^ They play a key role in cellular metabolism through their peptidyl‐prolyl cis‐trans isomerase (PPIase) activity, which catalyzes a rate‐limiting step in protein folding.^1^, ^2^, ^3^, ^4^ First identified in 1984 as high‐affinity binding proteins for the immunosuppressant cyclosporin A (CsA), cyclophilins have more recently been recognized as potential panallergens, a role that has long been overlooked in the context of allergy diagnosis.^4^, ^5^, ^6^, ^7^ Fungal cyclophilins, including Psi c 2 from Psilocybe cubensis, Asp f 11 from Aspergillus fumigatus, and Mala s 6 from Malassezia sympodialis, were among the first to be described as allergens.^8^, ^9^, ^10^ Subsequently, plant‐derived cyclophilins such as Ara h 18 (peanut), Bet v 7 (birch pollen), Cat r 1 (periwinkle), and Ole e 15 (olive pollen) have been identified, while homologues have also been detected in dust mites.^7^, ^11^, ^12^, ^13^, ^14^, ^15^ The growing recognition of cyclophilins as IgE‐binding proteins underscores a growing awareness of their allergenic potential.

In several respects, cyclophilins resemble profilins, a well‐established family of panallergens. Both are highly conserved proteins distributed across plants, fungi, and animals and both elicit broad IgE cross‐reactivity among unrelated species, leading to complex patterns of polysensitization.^5^, ^6^, ^10^, ^14^, ^16^, ^17^ Despite being considered “minor” allergens, sensitization to cyclophilins, similar to other plant panallergens such as profilins, may represent a marker of advanced and long‐lasting allergic sensitization associated with molecular spreading, while their direct clinical relevance remains uncertain.^5^, ^10^ Yet, in contrast to profilins such as Bet v 2 and Phl p 12, which are extensively characterized and routinely included in molecular allergy diagnostics, cyclophilins like Bet v 7 and Ara h 18 remain insufficiently studied and are not widely available in current specific IgE (sIgE) diagnostic panels.^5^ This discrepancy reveals a notable gap in our understanding of their clinical significance.

Several key questions remain unresolved. The biological role of cyclophilins within pollen and their contribution to allergenicity are not fully understood.^11^, ^12^, ^18^ It is unclear to what extent their intrinsic protein function influences their allergenic potential, and the precise cross‐reactivity patterns among cyclophilins from diverse sources (plants, molds, animals) have not yet been comprehensively defined, with only fragmentary structural data available for plant cyclophilins.^13^, ^14^ Furthermore, although cyclophilins are stress‐inducible proteins, their possible involvement in sensitization processes, particularly in the context of environmental modifiers such as pollution, remains to be elucidated.^11^, ^13^, ^18^ Finally, their clinical impact may be underestimated, as current diagnostic tools do not adequately capture their contribution to polysensitization.^5^

Against this background, the present study aims to provide a functional characterization of the birch pollen cyclophilin Bet v 7 and to assess its cross‐reactivity with the peanut cyclophilin Ara h 18. These two molecules were selected because Bet v 7 represents a well‐characterized pollen cyclophilin with documented IgE reactivity, while Ara h 18 is a food‐derived cyclophilin implicated in pollen–food allergy syndrome (PFAS).^11^, ^19^ Moreover, Bet v 7 and Ara h 18 share a high degree of sequence and structural homology, making them ideal candidates to investigate IgE cross‐reactivity within the plant cyclophilin family.^16^ Both allergens were the only cyclophilins available in the validated ImmunoCAP assay platform, enabling reliable measurement of sIgE in sera. Finally, we explored the functional relevance of Bet v 7 by assessing its capacity to induce mast cell activation and its stability to heat and saliva exposure, thereby providing insight into its potential clinical relevance in children with seasonal allergic rhinitis (SAR).

## 2 METHODS

### 2.1 Study population

We examined 100 samples selected from the Panallergens in Pediatrics (PAN‐PED) cohort study, an Italian multicenter clinical survey of children aged 4–18 years with pollen‐induced SAR, with or without concomitant asthma, enrolled between 2009 and 2011. The PAN‐PED cohort study was first described and published by Dondi et al.^20^ In the present work, we analyzed samples from this cohort and provide a summary of the most relevant study characteristics. Patients were recruited from 16 pediatric outpatient clinics across 14 Italian cities.^20^ Eligibility criteria for the PAN‐PED study included: (1) age between 4 and 18 years; (2) a history of pollen‐induced allergic rhinoconjunctivitis in at least one of the two preceding pollen seasons, with or without concomitant asthma; and (3) a positive skin prick test to the relevant pollen extracts. Exclusion criteria were: (1) prior allergen immunotherapy (AIT) for any pollen allergen; and (2) any other severe chronic disease. Parents or guardians of all participants provided written informed consent. The PAN‐PED study received approval from the ethics committee of each participating center.^20^ For the purposes of the present study, 100 sera were non‐randomly selected from the original PAN‐PED cohort, corresponding to the two subgroups previously described by Matricardi et al.: (1) 74 children with IgE to birch pollen extract but without detectable IgE to Bet v 1, Bet v 2, or Bet v 4, and (2) 26 children with the highest IgE levels to Bet v 1.^12^ This subset was chosen to represent distinct birch pollen sensitization patterns and to cover a broad range of IgE reactivity to Bet v 7, ranging from <0.1 to 216.4 kU_A_/L. The selection was not intended to be epidemiologically representative of the full PAN‐PED cohort but was designed to facilitate molecular and functional analyses of cyclophilin sensitization.^12^, ^20^

### 2.2 Skin prick tests

Skin prick tests (SPT) were performed using a standard panel of commercial extracts (ALK‐Abelló, Milan, Italy), which included birch. Histamine 0.1 mg/ml solution served as the positive control, and glycerol solution as the negative control. Morrow‐Brown needles were used to prick the skin, and wheal reactions were read after 15 min. A wheal diameter of ≥3 mm after subtraction of the negative control was considered positive.

### 2.3 Recombinant cyclophilins and allergens

Recombinant (r)Bet v 7.0101 (Acc. No. CAC84116) and rAra h 18.0101 (Acc. No. XP_025675300) were expressed as hexahistidine tagged proteins in E. coli BL21‐AI and purified by immobilized metal ion affinity chromatography (IMAC) and size‐exclusion chromatography (SEC). Purity and homogeneity of the final preparations were assessed by SDS‐PAGE of reduced and non‐reduced samples and analytical SEC. No remaining host cell proteins could be observed in SDS‐PAGE, and the proteins produced a single, symmetrical peak in SEC. rBet v 1 was purchased from InBio and used as supplied.

### 2.4 IgE tests

Peripheral venous blood was taken at recruitment and the serum stored in aliquots at −70°C until use. ImmunoCAP fluorescence enzyme immunoassay (Thermo Fisher Scientific, Uppsala, Sweden) was used to determine total IgE (tIgE) and sIgE serum levels to various pollen allergen extracts and molecules. Commercial ImmunoCAP assays were used for the detection of sIgE to Bet v 1, Bet v 2, Bet v 4, Phl p 1, and Phl p 5. Measurement of sIgE to the cyclophilins Bet v 7 and Ara h 18 was performed using experimental ImmunoCAP assays employing recombinant allergens. Experimental ImmunoCAP tests were prepared as described and the absence of assay background and unspecific IgE binding was verified using a pool of negative sera, with or without addition of myeloma IgE to 3000 kU_A_/L.^21^ Results for all IgE tests were expressed in kU_A_/L and considered positive at ≥0.35 kU_A_/L.

### 2.5 IgE inhibition assays

IgE cross‐reactivity between Bet v 7 and Ara h 18 was assessed by ImmunoCAP inhibition assays using three selected sera (P1, P2, and P3). Serum samples (100 μl) were pre‐incubated for 60 min with a 100‐fold molar excess of rBet v 7 or rAra h 18 prior to measurement of sIgE binding to the respective and cross‐reactive allergens. Inhibition was expressed as the percentage reduction of IgE binding compared with non‐inhibited serum controls.

### 2.6 Mast cell activation test

**FIGURE 1:**
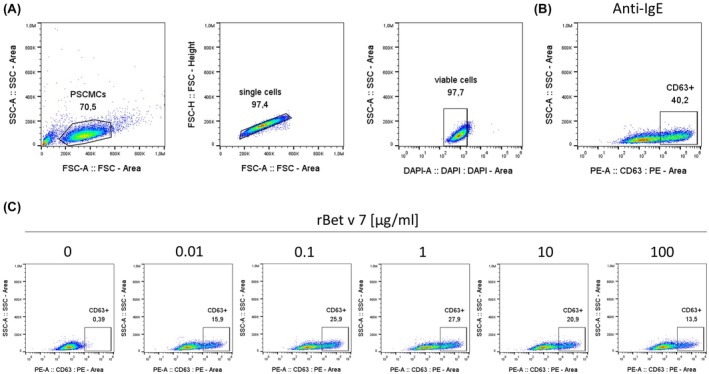
MAT gating strategy. Numbers above the gated regions represent the percentage of cells within the outlined area. (A) Representative flow cytometry plots of live PSCMCs. Cells were first gated based on side scatter area (SSC‐A) versus forward scatter area (FSC‐A) to exclude debris and aggregates. Singlets were then identified using FSC‐H versus FSC‐A, followed by exclusion of non‐viable cells using DAPI staining. (B) Representative flow cytometry plot of pre‐gated live PSCMCs showing CD63‐positive cells after stimulation with anti‐IgE. (C) Representative flow cytometry plots of pre‐gated PSCMCs sensitized with patient serum and subsequently stimulated with increasing concentrations of rBet v 7 (0–100 μg/ml), demonstrating dose‐dependent activation.

For the mast cell activation tests (MAT), sera from 11 children were selected from the PAN‐PED cohort, including individuals from both the predefined subset of 100 sera and the remaining cohort participants. These samples were chosen to represent a broad range of Bet v 7–specific IgE levels, including both sensitized and non‐sensitized individuals, in order to assess the functional activity of rBet v 7–induced mast cell activation. The MAT was conducted using a 96‐well format, with 50,000 primary human mast cells from peripheral blood precursors (PSCMCs) per well. PSCMCs were differentiated and cultured according to the protocol described by Schmetzer et al.^22^ For passive sensitization, PSCMCs were plated into wells and incubated with purified human myeloma IgE (Millipore, 1 μg/ml) or patient serum (10% diluted in medium) at 37°C and 5% CO_2_ overnight. Cells were then washed with Dulbecco's Phosphate‐Buffered Saline (DPBS) and incubated for 1 h with medium as a negative control, polyclonal rabbit anti‐human IgE (Bethyl) (1 ng/μl) as a positive control, and varying concentrations of rBet v 7 (0.01 μg/ml, 0.1 μg/ml, 1 μg/ml, 10 μg/ml, and 100 μg/ml). After stimulation, cells were washed and incubated for 5 min with 30 μl TruStain FcX (BioLegend) at room temperature (RT), followed by a 20‐min incubation with anti CD63‐PE (clone H5C6, diluted 1:100 in DPBS). Afterwards, the cells were washed again and stained with DAPI prior to measurement. Flow cytometric measurements were performed on the spectral cell analyzer (ID7000, Sony) and analyzed using the gating strategy shown in Figure 1. MAT results were calculated as the percentage of CD63‐positive cells and are expressed as the percentage of activated PSCMCs.

### 2.7 Heat and saliva treatment of allergen

Prior to stimulation in the MAT, rBet v 1 was subjected only to heat treatment at 60°C, whereas rBet v 7 was subjected to both heat and saliva treatment. For heat treatment, rBet v 7 was incubated at 60°C, 80°C, or 100°C for 10 min in DPBS. For saliva treatment, a 2 μg/ml solution of rBet v 7 was mixed 1:2 with freshly collected human saliva or saliva supplemented with trypsin (0.05%, Bio&Sell) and incubated at 37°C for 5 min. Following treatment, the samples were immediately used for stimulation in the MAT.

### 2.8 Sequence alignment and structural modeling of Bet v 7 and Ara h 18

Pairwise amino acid sequence alignment of Bet v 7 (Acc. No. CAC84116) and Ara h 18 (Acc. No. XP_025675300) was performed using NCBI BLASTp and visualized with NCBI Multiple Sequence Alignment Viewer (version 1.26.0).^23^

Three‐dimensional structures of Bet v 7 (UniProt Q8L5T1) and Ara h 18 (UniProt A0A6B9VF68) were predicted using AlphaFold2, and structural superposition was performed using TM‐align to assess conformational similarity, with TM‐scores calculated to quantify structural homology.^24^, ^25^

### 2.9 Statistics

Age was reported as mean ± SD; categorical data as *n* (%). Between‐group comparisons used chi‐squared or *t*‐tests. Correlations between sIgE levels (Bet v 7 vs. Ara h 18, Bet v 7 vs. activated PSCMC percentages) were assessed using Pearson's correlation coefficient on log‐transformed data, with significance at *α* = 0.05 and 0.01 (95% CIs via Fisher's *z*‐transformation). For heat and saliva experiments, normality was assessed using Shapiro–Wilk tests (*α* = 0.05). Non‐normally distributed data were compared using Mann–Whitney U tests with Cliff's delta effect sizes; results reported as median (IQR). For the heat experiment specifically, overall differences between temperature conditions were evaluated using the Friedman test (non‐parametric repeated measures). Pairwise comparisons employed Wilcoxon signed‐rank tests, with effect sizes calculated using Cohen's *d* for paired samples. Statistical significance was set at *p* < 0.05. Analyses used Stata 16.1.

## 3 RESULTS

### 3.1 Characteristics of the study population subsets

Among the 1263 patients enrolled in the PAN‐PED cohort, sera from 100 Italian children were non‐randomly selected according to predefined molecular sensitization patterns, as previously described, and all had a positive SPT to birch pollen extract.^12^ This subset was selected to assess the relationship between serum IgE reactivity to Bet v 7 and Ara h 18. The two groups were comparable with respect to sex, age distribution, SAR duration, and asthma prevalence. However, the 100‐patient subset displayed higher rates of anaphylaxis (10% vs. 6%) and atopic dermatitis (51% vs. 36%), as well as increased serum tIgE levels (824.0 vs. 371.5 kU/L), consistent with a more complex allergic phenotype (Table 1).

**TABLE 1:** Clinical and demographic characteristics of the PAN‐PED cohort (*n* = 1263) and the selected subset of patients (*n* = 100).

Characteristic	All	Included
No.	1263	100
Age (years)	10.5 (3.39)	9.6 (3.40)
Male sex	858 (68%)	75 (75%)
Age (years) at SAR onset	5.2 (2.82)	4.4 (2.58)
SAR duration (years)	5.2 (3.33)	5.3 (3.31)
Moderate‐to‐severe SAR (ARIA)	656 (52%)	53 (53%)
Asthma	504 (40%)	41 (41%)
OAS	311 (25%)	37 (37%)
Anaphylaxis	73 (6%)	10 (10%)
Urticaria‐angioedema	264 (21%)	24 (24%)
AD	453 (36%)	51 (51%)
Allergic GI symptoms	85 (7%)	12 (12%)
Total IgE serum levels (kU/L)	371.5 (2.95)	824.0 (2.69)
IgE to Cyp (Bet v 7)	ND	55 (55%)
IgE to Cyp (Ara h 18)	ND	55 (55%)
IgE to PR‐10 (Bet v 1)	ND	26 (26%)
IgE to profilin (Bet v 2)	ND	6 (6%)
IgE to polcalcin (Bet v 4)	ND	1 (1%)
IgE to group 1 grass allergen (Phl p 1)	ND	68 (68%)
IgE to group 5 grass allergen (Phl p 5)	ND	46 (46%)

*Note*: Clinical characteristics are presented descriptively to provide context for the selected sera. The subset of 100 sera was non‐randomly selected based on predefined molecular sensitization patterns and is therefore not intended for epidemiological or inferential statistical comparison with the full cohort.^12^ Data are presented as numbers (%) or mean (SD) for all characteristics, except for total IgE serum levels, which are expressed as geometric mean (SD).

Abbreviations: AD, atopic dermatitis; ARIA, Allergic Rhinitis and Its Impact on Asthma; GI, gastrointestinal; ND, not determined; OAS, oral allergy syndrome; SAR, seasonal allergic rhinitis.

### 3.2 Levels of IgE to rBet v 7 strongly correlate with Ara h 18

**FIGURE 2:**
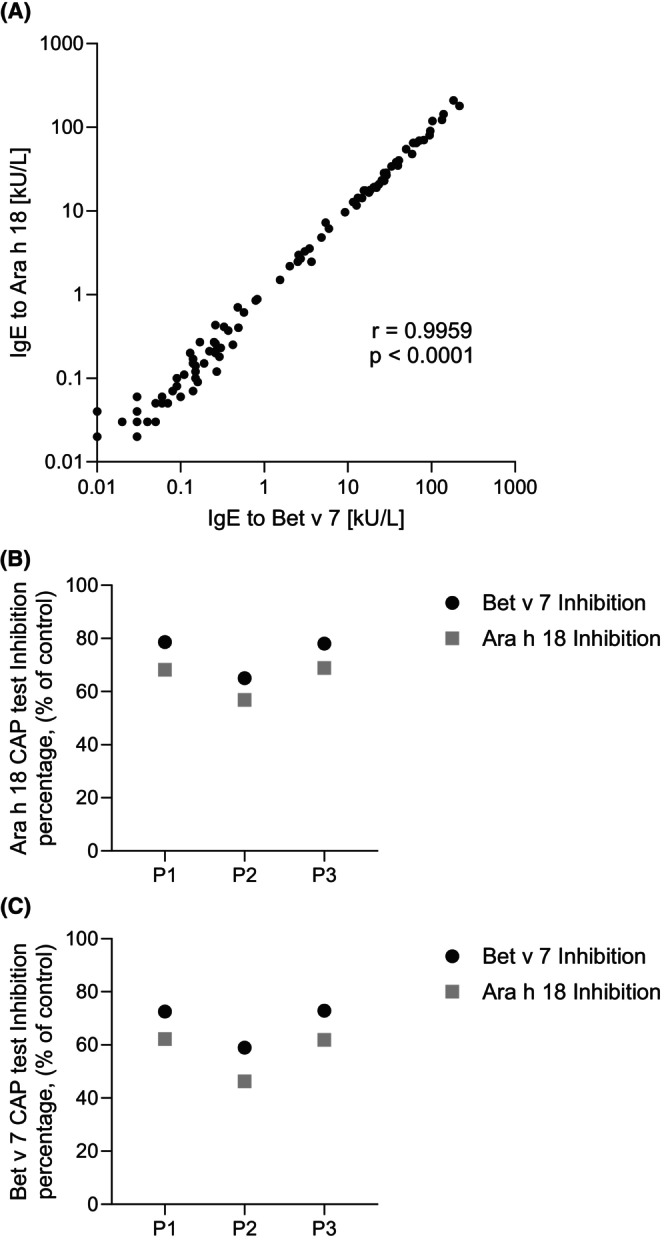
Correlation and IgE cross‐inhibition between Bet v 7 and Ara h 18. (A) Relationship between the serum levels of IgE to Bet v 7 vs. IgE to Ara h 18 in 100 Italian children with SAR. Each point represents an individual patient. Specific IgE levels are expressed in kU_A_/L on a logarithmic scale. Using Pearson's correlation analysis, a strong positive correlation was found between IgE to Bet v 7 and IgE to Ara h 18 (*r* = 0.9959, *r* ^2^ = 0.9919, 95% CI: 0.9940–0.9973, *p* < 0.0001). Three selected sera (P1, P2, and P3) were pre‐incubated with recombinant Ara h 18 (B) or Bet v 7 (C) prior to measurement of specific IgE binding to the allergens by ImmunoCAP. Results are expressed as percentage inhibition relative to non‐inhibited serum controls and demonstrate strong bidirectional IgE cross‐reactivity between both cyclophilins.

In the 100 selected sera, sIgE to Bet v 7 ranged from <0.1 to 216 kU_A_/L (median 0.81; mean 19.97 kU_A_/L), following a right‐skewed, log‐normal distribution. A strong positive correlation was observed between sIgE to Bet v 7 and Ara h 18 (*r* = 0.9959, *r* ^2^ = 0.9919, 95% CI: 0.9940–0.9973, *p* < 0.0001) (Figure 2A). This close relationship is supported by the high sequence and structural similarity between Bet v 7 and Ara h 18, both belonging to the cyclophilin family, reinforcing the concept of strong immunological cross‐reactivity.^11^

### 3.3 IgE cross‐reactivity between Bet v 7 and Ara h 18

To further assess IgE cross‐reactivity, inhibition experiments were performed in both directions using three selected sera (P1‐P3; Table 2). Pre‐incubation with rBet v 7 strongly inhibited IgE binding to Ara h 18, and conversely, Ara h 18 efficiently inhibited IgE binding to Bet v 7 (Figure 2B,C), confirming extensive bidirectional cross‐reactivity between these two cyclophilins. The high degree of mutual inhibition is consistent with their strong sequence and structural homology.

### 3.4 IgE to rBet v 7 induces mast cell activation in a dose‐dependent manner

**FIGURE 3:**
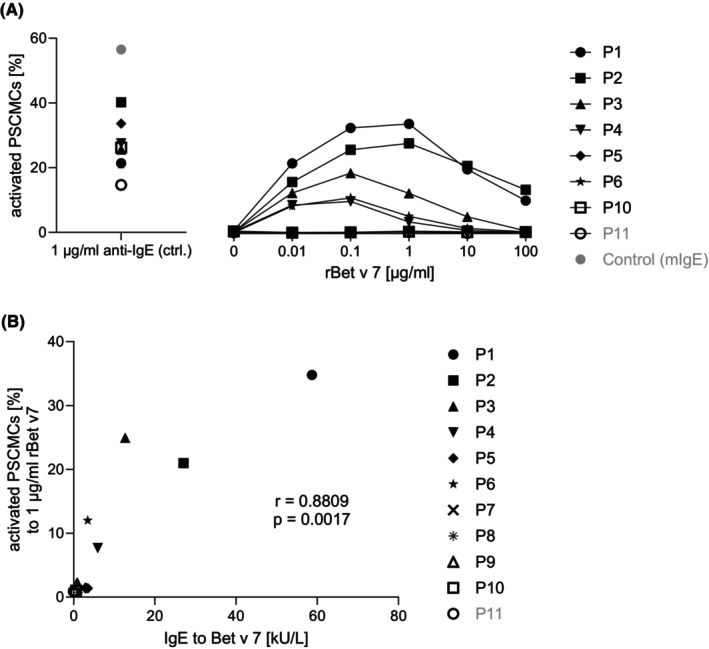
Mast cell activation with increasing concentrations of rBet v 7 and its correlation with specific IgE to Bet v 7. (A) Mast cell activation after sensitization with patient serum and stimulation with increasing concentrations of rBet v 7. PSCMCs were sensitized with sera from 8 patients (P1–P8), while P9–P11 could not be measured due to shortage of serum. Cells were then stimulated with rBet v 7 at concentrations ranging from 0 to 100 μg/ml. Activation was assessed by CD63 surface expression and is presented as percentage of activated mast cells. Data represent results from a single representative experiment. A dose‐dependent response was observed, with peak activation at intermediate concentrations and reduced responses at higher concentrations, consistent with a bell‐shaped dose–response curve. (B) Correlation between serum levels of specific IgE to Bet v 7 and the peak mast cell activation induced by rBet v 7 (1 μg/ml) in the presence of patient serum. Mast cell activation was quantified by CD63 expression and is shown as percentage of activated cells. Data on mast cell activation show mean values from three independent experiments. Pearson's correlation analysis revealed a strong positive relationship between serum IgE to Bet v 7 and mast cell activation (*r* = 0.8809, *r* ^2^ = 0.7760, 95% CI: 0.5225–0.9748, *p* = 0.0017).

To assess the functional relevance of IgE to Bet v 7, sera from 11 patients, selected to represent a broad range of Bet v 7–specific IgE levels including one negative control (P11), were analyzed in MAT experiments (Table 2). Due to limited serum availability, not all samples could be included in every experiment. Relevant mast cell activation (10%–35%) was observed in five of the eight evaluable patients, with responses increasing in a dose‐dependent manner, peaking at 0.1–1 μg/ml, and declining at higher allergen concentrations (Figure 3A). Three patients showed no or minimal activation.

**TABLE 2:** Clinical and demographic characteristics of the 11 patients included in the MAT experiments.

Characteristic/Patient No.	P1	P2	P3	P4	P5	P6	P7	P8	P9	P10	P11
Age (years)	9	8	10	10	4	11	8	15	17	4	12
Sex	M	M	F	M	M	F	M	M	F	M	F
Asthma	Y	‐	Y	Y	‐	Y	Y	‐	Y	Y	‐
OAS	‐	‐	Y	‐	Y	‐	Y	‐	Y	‐	‐
Anaphylaxis	‐	‐	‐	Y	‐	‐	‐	‐	‐	Y	‐
Urticaria‐angioedema	‐	‐	Y	‐	‐	‐	‐	‐	Y	‐	‐
AD	‐	‐	Y	‐	‐	Y	Y	‐	Y	Y	‐
Allergic GI symptoms	‐	‐	‐	‐	‐	‐	Y	‐	‐	Y	‐
SPT wheal reaction to birch extract (mm)	4.0	2.5	5.5	2.0	4.5	4.0	10.0	1.5	7.0	7.0	0.0
Total serum IgE levels (kU/L)	198.3	1223	312	395	2863	169	688	103	1249	1093	73.4
IgE to Bet v 7 (kU_A_/L)	58.70	27.05	12.69	5.93	3.48	3.47	1.92	1.06	0.86	0.57	0.1
IgE to Ara h 18 (kU_A_/L)	48.04	28.45	11.67	6.13	2.94	3.54	ND	ND	ND	0.61	ND
IgE to Bet v 1 (kU_A_/L)	0.1	0.1	0.1	0.1	0.2	0.1	43.0	0.1	84.2	138.0	0.1
IgE to Bet v 2 (kU_A_/L)	0.1	0.1	0.1	0.1	0.2	0.1	0.1	ND	0.1	7.1	ND
IgE to Bet v 4 (kU_A_/L)	0.1	0.1	0.1	0.1	0.3	0.1	0.1	ND	0.1	0.1	ND
IgE to Phl p 1 (kU_A_/L)	ND	83.5	32.4	5.8	270.0	0.1	ND	0.1	10.7	82.1	0.4
IgE to Phl p 5 (kU_A_/L)	ND	78.4	0.7	0.1	141.0	0.4	ND	5.0	0.1	48.2	0.1

*Note*: Sera were selected from the PAN‐PED cohort to cover a broad range of Bet v 7 sensitization and were sorted in descending order by Bet v 7–specific IgE levels. One Bet v 7–negative serum (P11) was included as a negative control and is marked in gray.

Abbreviations: “‐”, no; AD, atopic dermatitis; F, female; GI, gastrointestinal; M, male; ND, not determined; OAS, oral allergy syndrome; SPT, skin prick test; Y, yes.

### 3.5 Strong correlation between Bet v 7–specific IgE and mast cell activation

The association between Bet v 7–specific IgE levels and functional responses was analyzed to investigate the factors underlying variability in mast cell activation. Importantly, although Bet v 7–specific IgE represented only a minor fraction of tIgE in most individuals, a strong correlation was detected between sIgE levels to Bet v 7 and the percentage of activated PSCMCs (*r* = 0.8809, *r* ^2^ = 0.7760, 95% CI: 0.5225–0.9748, *p* = 0.0017) (Figure 3B), remaining statistically significant at both the 0.05 and 0.01 significance levels (critical values: ±0.666 and ±0.798, respectively for degrees of freedom equal to 7). Notably, patients with absent or marginal mast cell activation had sIgE concentrations to Bet v 7 around or below 3.5 kU_A_/L (Table 2). These findings indicate that, despite representing only a small proportion of tIgE and within a background of complex polysensitization, cyclophilin‐specific IgE can elicit functional mast cell activation in a subset of sensitized individuals.

### 3.6 rBet v 7 mediated mast cell activation is mild heat and saliva resistant

Heat and saliva pre‐treatments were performed to evaluate the stability of rBet v 7 under relevant digestive and thermal stress conditions. Saliva treatment was first used to simulate early digestive exposure and assess the resistance of rBet v 7 to salivary components.

**FIGURE 4:**
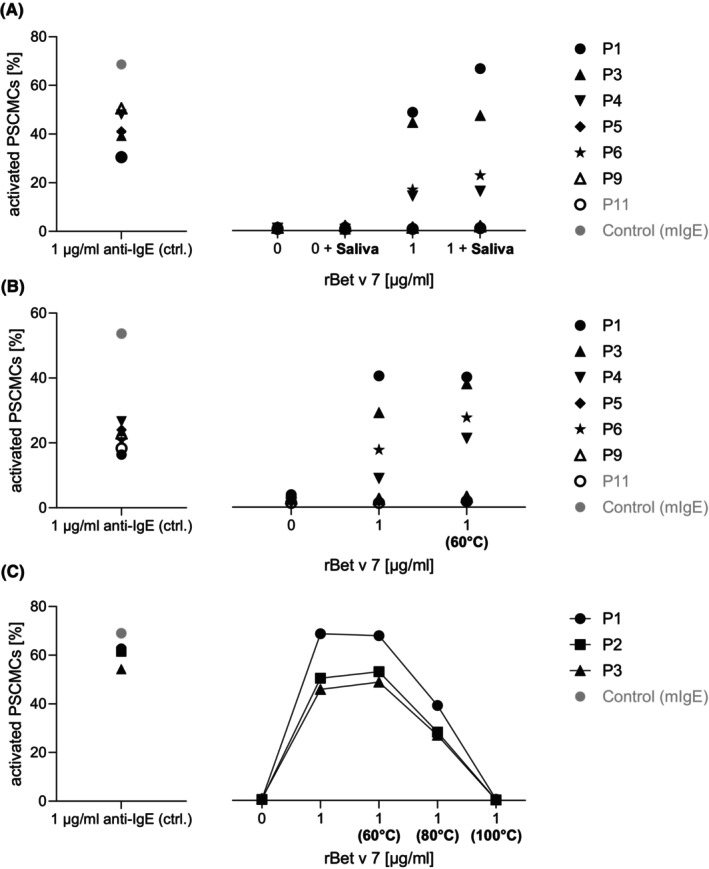
Mast cell activation induced by rBet v 7 with or without pre‐treatment. Mast cells were sensitized with sera of seven patients and stimulated with optimal concentrations of rBet v 7 (1 μg/ml), either untreated, incubated with human saliva (A), or heat‐treated (60°C, 10 min) (B). Patients P6, P8, and P10–P11 were not included due to shortage of serum. Additionally, mast cells were sensitized with sera from three patients and stimulated with optimal concentrations of rBet v 7 (1 μg/ml), either untreated or heat‐treated at 60°C, 80°C, or 100°C for 10 min (C). Activation was assessed by CD63 expression and is shown as percentage of activated cells. Data represent results from a single representative experiment. Mild heat and saliva exposure did not reduce mast cell activation, whereas higher temperatures resulted in a progressive reduction, most pronounced at 100°C.

For saliva treatment, Shapiro–Wilk tests indicated non‐normal distributions in both the rBet v 7 stimulated group without saliva treatment (W = 0.7754, *p* = 0.0155) and the group with rBet v 7 stimulation following saliva treatment (W = 0.8004, *p* = 0.0289). Median values were 8.005 (IQR: 22.637) and 9.185 (IQR: 27.177), respectively, with an absolute difference of 1.180 (14.7% relative difference). Mann–Whitney U test showed no statistically significant difference between groups (U = 25, *p* = 0.5054) with a small effect size (Cliff's delta = −0.219) (Figure 4A). The addition of trypsin to saliva, however, reduced mast cell activation by rBet v 7, confirming its sensitivity to enzymatic breakdown (data not shown).

Heat treatment was subsequently used to evaluate the thermal stability of rBet v 7 under relevant thermal stress conditions. For the heating experiment, Shapiro–Wilk tests indicated non‐normal distributions in both the rBet v 7 stimulated group without heat treatment (W = 0.8130, *p* = 0.0394) and the group with rBet v 7 stimulation following heat treatment (W = 0.8131, *p* = 0.0394). Descriptive analysis showed median values of 5.715 (IQR: 18.402) for the rBet v 7 stimulated group without heat treatment and 12.190 (IQR: 28.093) for the rBet v 7 stimulated group with heat treatment, representing an absolute difference of 6.475 and a relative difference of 113.3%. Mann–Whitney U test demonstrated no statistically significant difference between groups (U = 30, *p* = 0.8785). The effect size was negligible (Cliff's delta = −0.062), indicating minimal practical difference between the two groups despite the observed numerical difference in medians (Figure 4B). In contrast, heat treatment of rBet v 1 at 60°C resulted in reduced mast cell activation, in line with its known heat sensitivity (data not shown).^26^ To better mimic food‐processing conditions, rBet v 7 was additionally subjected to heat treatment at 80°C and 100°C. While heating at 60°C did not markedly reduce mast cell activation, exposure to higher temperatures resulted in a progressive reduction of allergenic activity, although residual activity at 80°C remained detectable (Figure 4C). Due to limited serum availability, these experiments were performed using three Bet v 7–positive sera. Consistently, a Friedman test revealed a significant overall effect of temperature on mast cell activation (*χ* ^2^ = 8.20, *p* = 0.042). Pairwise Wilcoxon signed‐rank tests showed no significant differences between individual conditions (all *p* ≥ 0.25), reflecting the limited statistical power with *n* = 3. However, effect sizes were consistently large for all comparisons involving thermal treatment at 80°C and 100°C (Cohen's *d* > 4.0), indicating substantial biological effects.

### 3.7 Bet v 7 and Ara h 18 share high sequence and structural homology

Pairwise sequence alignment of Bet v 7 and Ara h 18 revealed a high degree of amino acid conservation, with 155 of 171 residues being identical (91%), indicating strong primary sequence homology (Figure S1). Consistently, structural modeling using AlphaFold2 and subsequent structural superposition demonstrated nearly identical three‐dimensional conformations of both proteins, reflected by a very high TM‐score of 0.99346 (normalized by the length of Bet v 7), confirming their close structural similarity (Figure S2).

## 4 DISCUSSION

In this study of Italian children with SAR, we investigated the functional characteristics of IgE directed against Bet v 7, a member of the cyclophilin panallergen family. In our cohort, sIgE levels to Bet v 7 showed a strong correlation with those to Ara h 18, the peanut cyclophilin, and were associated with the ability of patient sera to induce mast cell degranulation in response to rBet v 7 stimulation. Importantly, rBet v 7 retained functional activity after exposure to mild heat and saliva. To our knowledge, this is the first study demonstrating that a plant‐derived cyclophilin can induce mast cell activation in a dose‐dependent manner using human stem cell–derived mast cells. Using this system, we show that very low concentrations of rBet v 7 (as low as 10 ng/ml) are sufficient to induce robust mast cell activation, and that the magnitude of this response correlates well with serum levels of Bet v 7–specific IgE. This high sensitivity contrasts with previous reports employing skin prick testing or RBL assays, which required substantially higher allergen concentrations and did not show clear correlations with IgE levels.^13^, ^18^ These findings provide novel insights into the biological and clinical relevance of cyclophilin sensitization.

The almost perfect correlation between IgE levels to Bet v 7 and Ara h 18 is consistent with their high sequence identity (91%) and implies high cross‐reactivity.^12^, ^16^, ^17^ Notably, this correlation was reciprocal, as no discordant cases were observed: all sera positive for Bet v 7 were also positive for Ara h 18, and vice versa. While this finding demonstrates nearly complete co‐sensitization to both cyclophilins, it does not allow identification of the primary sensitizing source. Addressing this question would require longitudinal studies and/or cohorts with distinct environmental exposure profiles. Reciprocal IgE inhibition experiments further confirmed this extensive cross‐reactivity, showing strong bidirectional inhibition of IgE binding and indicating near‐complete immunological cross‐recognition of both cyclophilins.

Similar levels of sequence identity are also observed among other plant cyclophilins.^16^, ^17^ Accordingly, the strong bidirectional IgE cross‐reactivity observed between Bet v 7 and Ara h 18 is in line with previous reports demonstrating widespread cross‐reactivity among plant cyclophilins.^17^ In contrast, Fujita et al. reported limited cross‐reactivity between Bet v 7 and carrot cyclophilin, indicating that sequence divergence among plant cyclophilins can result in variable IgE cross‐recognition.^7^ Together, these findings suggest that the extent of cross‐reactivity depends on the degree of structural conservation among individual cyclophilin family members.

Our results further suggest that mast cell activation triggered by rBet v 7 is likely to be mirrored by other plant cyclophilins, including Ara h 18. Interestingly, most PAN‐PED participants sensitized to Bet v 7 were negative for Bet v 1, the major birch allergen, but positive for Phl p 1 and/or other major pollen allergens.^12^ This points towards intermolecular epitope spreading from cyclophilin‐containing pollen (grass, olive, plane tree, etc.) to Bet v 7 and Ara h 18.^12^ Indeed, the initial identification of Ara h 18 was linked to peanut‐sensitized patients without detectable IgE to other known Ara h components but sensitized to pollen.^16^

Conversely, cross‐recognition with cyclophilins from non‐plant sources such as bacteria, fungi, arthropods, or mammals, which share lower sequence homology, appears limited, and no autoreactive phenomena were observed in our Bet v 7–sensitized cohort.^13^ In contrast, sensitization to the fungal cyclophilin Mala s 6 from Malassezia sympodialis has been linked to severe atopic dermatitis.^27^

Our results suggest that the cyclophilin panallergen family represents a useful model to study IgE cross‐reactivity to panallergens. In particular, allergens with higher sequence and structural similarity to the human counterpart may provide insight into mechanisms potentially linking allergic sensitization and autoimmune phenomena, although no evidence of autoreactivity was observed in our cohort.^27^ On the other hand, our observations also have implications for the design and use of in vitro diagnostic tests to detect serum sIgE to cyclophilin. Considering the almost identical measurements of sIgE levels to Bet v 7 and to Ara h 18, we suggest that one member of the plant cyclophilin family could function as a surrogate for all plant cyclophilins in singleplex or multiplex sIgE tests. Broader studies are needed to validate this hypothesis.

The bell‐shaped dose–response curve observed in MAT, with reduced degranulation at high rBet v 7 concentrations, is consistent with established models of antigen‐FcεRI interactions, where excess allergen saturates IgE without promoting cross‐linking.^28^ Similar patterns have been reported in other MAT settings.^29^, ^30^ Additional mechanisms such as receptor internalization or negative feedback regulation, as described in basophils, may also contribute.^31^, ^32^, ^33^

Clinically, the survey at the basis of our present study identified 10 SAR patients sensitized to Bet v 7 but negative for profilins, PR‐10, and nsLTPs, none of whom had oral allergy syndrome at the time of sampling.^12^ This observation questions the direct clinical impact of cyclophilin‐specific IgE, despite their ubiquity in fruits and vegetables. Nevertheless, our MAT findings suggest that Bet v 7 sensitization may contribute to peanut reactivity via cross‐recognition with Ara h 18, raising the possibility of symptom development over time. Moreover, the PAN‐PED survey initially found an association between Bet v 7 sensitization and asthma comorbidity; however, this association was no longer observed after multivariate adjustment.^12^ Overall, these data suggest that cyclophilin‐specific IgE is a minor but potentially contributing factor within complex polysensitization patterns, and that clinical implications remain uncertain.

We also observed that rBet v 7 retained its ability to induce mast cell activation after mild heat and saliva exposure, conditions chosen to reflect early‐phase oral contact relevant to PFAS. Notably, heat treatment at 60°C did not reduce mast cell activation, with rBet v 7 remaining fully capable of inducing robust degranulation. Since saliva contains proteolytic enzymes, we additionally performed trypsin digestion as a representative serine protease control, which reduced mast cell activation, demonstrating that rBet v 7 is not generally protease resistant.^34^ These findings indicate that Bet v 7 may remain functionally active during oral exposure while being susceptible to stronger enzymatic digestion. Simulated gastric fluid assays were beyond the scope of this study, which focused on early oral‐phase mechanisms rather than gastrointestinal food allergy and are not readily compatible with mast cell activation assays due to impaired mast cell viability.

Although heating at 60°C alone does not fully reflect typical food‐processing conditions, this temperature was included for comparison, as it approximates the melting temperature of Bet v 1.^35^ Extending the heat treatment to 80°C and 100°C resulted in a progressive reduction of rBet v 7 allergenic activity, indicating partial thermal stability with residual activity at intermediate temperatures. These findings suggest that Bet v 7 may retain allergenic potential during mild thermal processing but becomes increasingly inactivated under more intense heat exposure, supporting its potential relevance in early oral exposure rather than systemic food allergy.

Several limitations should be acknowledged. First, we examined only Bet v 7 in MAT and correlated sIgE levels to Ara h 18; cyclophilins from other plants may display distinct features despite sequence homology. Second, we did not investigate cyclophilins from mites, fungi, mammals, or humans, so potential cross‐reactivity with these homologues cannot be entirely excluded. Third, we relied on MAT and did not perform basophil activation tests (BAT), leaving open the question of whether our findings are fully reproducible in basophils.

In conclusion, we demonstrate that sera from Bet v 7–sensitized children with SAR recognize Ara h 18 and that rBet v 7 can induce mast cell degranulation in vitro. These findings provide novel insights into the biology of cyclophilins, strengthen the case for their inclusion in molecular diagnostic testing, and open the way for broader studies to define their clinical relevance across plant and non‐plant sources in allergic disease.

## AUTHOR CONTRIBUTIONS

**Wilhelm Sponholz:** Conceptualization; formal analysis; visualization; writing – original draft; investigation; validation; methodology. **Carolin Steinert:** Conceptualization; visualization; methodology; writing – review and editing; validation. **Francesco Monnati:** Formal analysis; writing – review and editing; validation. **Ekaterina Potapova:** Project administration; writing – review and editing. **Jonas Lidholm:** Investigation; writing – review and editing; validation; resources. **Lars Mattsson:** Investigation; writing – review and editing; validation; resources. **Enrico Scala:** Writing – review and editing; resources. **Roberto Bernardini:** Writing – review and editing; resources. **Carlo Caffarelli:** Writing – review and editing; resources. **Antonella Casani:** Writing – review and editing; resources. **Rosa Cervone:** Writing – review and editing; resources. **Elisabetta Del Duca:** Writing – review and editing; resources. **Pasquale Comberiati:** Writing – review and editing; resources. **Giovanna De Castro:** Writing – review and editing; resources. **Michele Miraglia Del Giudice:** Writing – review and editing; resources. **Iride Dello Iacono:** Writing – review and editing; resources. **Andrea Di Rienzo Businco:** Writing – review and editing; resources. **Marcella Gallucci:** Writing – review and editing; resources. **Arianna Giannetti:** Writing – review and editing; resources. **Viviana Moschese:** Writing – review and editing; resources. **Elena Varin:** Writing – review and editing; resources. **Annamaria Bianchi:** Writing – review and editing; resources. **Mauro Calvani:** Writing – review and editing; resources. **Tullio Frediani:** Writing – review and editing; resources. **Francesco Macrì:** Resources; writing – review and editing. **Nunzia Maiello:** Writing – review and editing; resources. **Francesco Paravati:** Writing – review and editing; resources. **Umberto Pelosi:** Writing – review and editing; resources. **Diego Peroni:** Writing – review and editing; resources. **Giuseppe Pingitore:** Writing – review and editing; resources. **Mariangela Tosca:** Writing – review and editing; resources. **Anna Maria Zicari:** Writing – review and editing; resources. **Giampaolo Ricci:** Writing – review and editing; resources. **Riccardo Asero:** Writing – review and editing; resources. **Salvatore Tripodi:** Writing – review and editing; resources. **Paolo Maria Matricardi:** Conceptualization; supervision; project administration; writing – review and editing; methodology; validation; resources. **Joerg Scheffel:** Writing – review and editing; conceptualization; supervision; methodology; project administration; validation; resources.

## FUNDING INFORMATION

Paolo Maria Matricardi is funded by the Deutsche Forschungs Gesellschaft (MA 4720/2; DFG, Bonn, Germany). Enrico Scala was funded, in part, by the Italian Ministry of Health, Current Research Program 2018–2020. Reagents for the study have been kindly provided by ALK‐Abelló (skin prick tests) and Thermo Fisher Scientific (all IgE tests; rBet v 7 and rAra h 18). Carolin Steinert and Wilhelm Sponholz are funded by the Deutsche Forschungsgemeinschaft (DFG, German Research Foundation) as part of the clinical research unit (CRU339): Food allergy and tolerance (FOOD@) – 409525714.

## CONFLICT OF INTEREST STATEMENT

JL and LM are employees of Thermo Fisher Scientific. ES has received consultant arrangements and speakers' bureau participation from Stallergenes and Thermo Fisher Scientific. JS has no conflict of interest regarding this manuscript. Outside of this work, he has conducted studies for or received research funds from Allakos, Ascilion, AstraZeneca, Attovia, Aquestive, Beiersdorf, CSL Behring, Celldex, Escient, Evommune, Genentech, Invea, Jasper, Novartis, Sanofi, Servier, Septerna, and ThirdHarmonicBio. All the other authors declared no conflicts of interest in relation to the present study.

## Supporting information


Figure S1.

Figure S2.
