# Supplementary material for: IgE to cyclophilin Bet v 7 triggers mast cell activation and mediates cross‐reactivity with Ara h 18 in children with seasonal allergic rhinitis
Source: Pediatr Allergy Immunol. 2026 Mar 4;37(3):e70308. doi: 10.1111/pai.70308 (PMC12960291; doi:10.1111/pai.70308)
Supplement: Supplementary file 1 — Figure S1. [file PAI-37-e70308-s001.pdf]

NCBI Multiple Sequence Alignment Viewer, Version 1.26.0

Sequence ID		Start	Alignment (1–171)	End	Organism
CAC84116	(+)	3	SNPKVFFDMEVGGQPVGRIVMELYADTTPRTAENFRALCTGEKGNGRSGKPLHYKKSSFHRVIPGFMCQGGDFTAGNGTGGESIYGAKFADENFIKKHTGPGILSMANAGPGTNGSQFFICTAKTEWLDGKHVVFGQVVEGLDIVKAIEKVGSSSGRTSKPVVVADCGQLS	173	Betula pendula
XP_025675300	(+)	2	ANPRVFFDMTIGGQPAGRIIMELFADTTPRTAENFRALCTGEKGVGRSGKPLHYKGSSFHRVIPNFMCQGGDFTAGNGTGGESIYGSKFADENFIKKHTGPGILSMANAGQNTNGSQFFICTAKTEWLDGKHVVFGQVVEGLDVVREIEKVGSSSGRTSKPVVVADCGQLS	172	Arachis hypogaea

Supplementary Figure S1: Pairwise amino acid sequence alignment of Bet v 7 and Ara h 18. The alignment confirms their high sequence identity (155/171 residues; 91%) between Bet v 7 (Acc. No. CAC84116) and Ara h 18 (Acc. No. XP_025675300), supporting their strong structural homology.
