# Supplementary material for: IgE to cyclophilin Bet v 7 triggers mast cell activation and mediates cross‐reactivity with Ara h 18 in children with seasonal allergic rhinitis
Source: Pediatr Allergy Immunol. 2026 Mar 4;37(3):e70308. doi: 10.1111/pai.70308 (PMC12960291; doi:10.1111/pai.70308)
Supplement: Supplementary file 2 — Figure S2. [file PAI-37-e70308-s002.pdf]

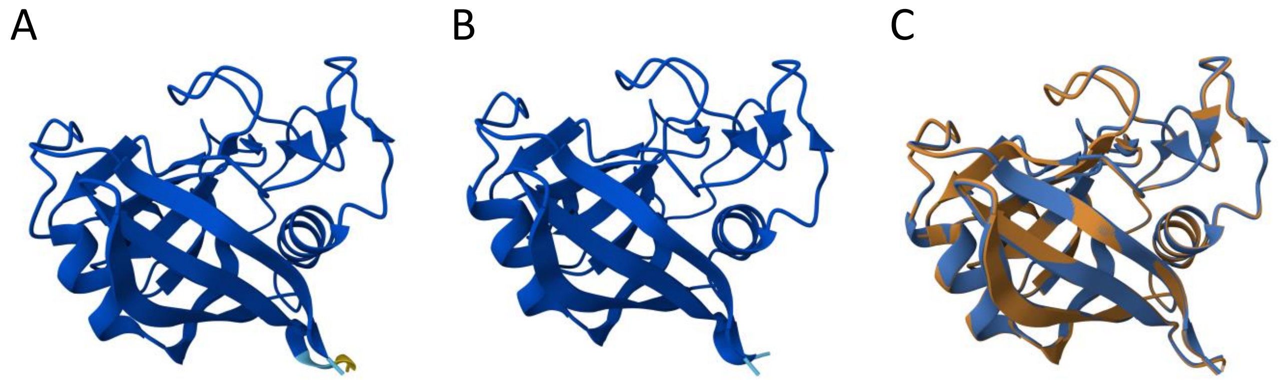

Supplementary Figure S2: Structural comparison of Bet v 7 and Ara h 18 based on AlphaFold2-predicted three-dimensional models. Bet v 7 (UniProt Q8L5T1) (A) and Ara h 18 (UniProt A0A6B9VF68) (B) are shown, respectively. Structural superposition (C) of both proteins, demonstrating very close conformational similarity (TM-score = 0.99346, normalized by the length of Bet v 7), consistent with their high structural homology.
